# Efficient encapsulation of CRISPR-Cas9 RNP in bioreducible nanogels and release in a cytosol-mimicking environment

**DOI:** 10.1186/s11671-025-04316-5

**Published:** 2025-07-26

**Authors:** Peter Westarp, Thorsten Keller, Jessica Brand, Sonja Horvat, Krystyna Albrecht, Chase Beisel, Juergen Groll

**Affiliations:** 1 Department of Functional Materials in Medicine and Dentistry, University of Wuerzburg, Wuerzburg, Germany; 2 Helmholtz Institute for RNA-Based Infection Research (HIRI), Wuerzburg, Germany

**Keywords:** CRISPR, Cas9, Nanogel, Nanoprecipitation, Nanoparticle, TXTL

## Abstract

CRISPR/Cas9-mediated programmable gene editing has disrupted the biotechnology industry since it was first described in 2012. Safe in vivo delivery is a key bottleneck for its therapeutic use. Viral vector-mediated delivery raises concerns due to immunogenicity, long-term expression, and genomic disruption. Delivery of pre-complexed ribonucleoprotein (RNP) reduces off-target effects, and recombinant Cas9 production is more cost-effective than viral vector synthesis. CRISPR-Cas RNPs do not possess intrinsic cell entry mechanisms, and physical delivery methods are confined to ex vivo editing, necessitating non-viral delivery approaches. Nanogels (NG) are biocompatible polymeric nanoparticles capable of entrapping proteins. Here, we report the first proof of principle that NGs from thiol-functionalized polyglycidol can entrap active RNPs with high efficiency (60 ± 2%). We call these particles CRISPR-Gels. A commercially available *E. coli* lysate for cell-free transcription and translation (TXTL) was used to mimic the intracellular reductive degradation of NGs while providing a real-time fluorescence readout of RNP activity. Degradation and RNP activity were observed within 30–90 min. The described TXTL assay can be utilized to evaluate the release of RNP in a cytosol-mimicking environment from redox-sensitive nanoparticles in a high-throughput and cost-effective way. Further studies are needed to assess the in vitro and in vivo performance of CRISPR-Gels.

**Supplementary Information:**

The online version contains supplementary material available at 10.1186/s11671-025-04316-5.

## Introduction

Clustered Regularly Interspaced Short Palindromic Repeats (CRISPR) sequences were first observed in 1987 in the *E. coli* genome [1]. Along with CRISPR-associated proteins (Cas), they form a bacterial immune system that fights off bacteriophage infections [2]. Cas9 can be utilized as a programmable nuclease. Guided by RNA, it can target almost any location within the genome [3]. Modified Cas enzymes have been developed, enabling a multitude of tasks beyond inducing double-strand breaks, such as single-strand breaks, precise point mutations (base editing), or transcription regulation. In the past decade, CRISPR systems have disrupted the field of genome editing. For example, by providing novel, faster, and more cost-effective methods to create animal models such as knockout mice, engineer crops, or perform genetic screens [4]. They also offer new strategies for treating genetic diseases in humans [5]. 

To this end human cells can be modified ex vivo (e.g. through electroporation) and then transplanted into the patient, or the CRISPR system can be delivered in vivo. Most clinical studies as well as the first FDA-approved CRISPR Therapy (Casgevy®, Vertex Pharmaceuticals) use the former approach [6, 7]. Ex vivo editing is highly specific (100%) and allows for phenotypic and genotypic cell-quality control before administration. However, this procedure is time and resource-intensive and limited to cell types that can be expanded in culture i.e. hematologic cells. In vivo, targeted delivery can be achieved through direct local administration of Cas9-RNP e.g. in the brain [8], skin [9], or solid tumors [10]. While this strategy mostly avoids editing in unintended tissues, the therapeutic effect is limited closely around the injection site and is practical only for organs accessible for direct injection.

These limitations can be overcome by systemic in vivo approaches. Even for hematopoietic cells in vivo editing might be preferential to reduce costs and potential changes in cellular phenotype from ex vivo culture. Systemic administration requires a delivery vehicle providing protection from degradation, evasion from the patient’s immune system, and the ability to target specific tissues or cells. The components of the CRISPR system can be delivered as DNA, mRNA, or the active RNP complex. Viral vectors like lentiviruses, adeno-associated viruses (AAVs), and adenoviruses (AdVs) are common and effective DNA-delivering vehicles, that had thousands of years to evolve under evolutionary pressure. Major safety concerns of viral delivery include immunogenicity, off-target effects due to long-term expression, and genomic integrity risks including insertional oncogenesis [11]. In contrast to DNA, RNP is degraded mostly within 24 h in cells, which can reduce off-target effects by limiting the exposure of the genome to the active genome-editing complex and reduce immunogenicity [12, 13]. Furthermore, recombinant Cas9 production in *E. coli* is scalable and cost-effective, compared to costly mammalian or insect cell culture for generating viral vectors [14]. Reflecting these advantages almost all clinical trials (ex vivo) have utilized Cas9 RNP or mRNA. Unlike naturally evolved viral tropisms, CRISPR-Cas RNPs and mRNAs lack inherent cell targeting and entry, requiring substantial engineering for systemic usage. To this end synthetic nanoparticles have been developed such as lipid nanoparticles (LNPs) [15–19], cationic polymers and peptides [20, 21] including a liposome templated nanogel [22], and gold nanoparticles [23]. Numerous review articles give a comprehensive overview of possible approaches. Common features of almost all synthetic delivery vehicles are size above 100 nm due to large CRISPR–Cas9 and positively charged surfaces to enhance cell entry and endosomal escape [24–27]. LNPs are the most widely used particles and were used in the first successful systemic in vivo delivery of Cas9 mRNA and sgRNA in humans for treating transthyretin amyloidosis [28]. In an active clinical trial (NCT05398029; Registration Date: 2022-05-31), patients with heterozygous familial hypercholesterolemia are being treated with LNPs containing Cas9 base editor mRNA and sgRNA to reduce PCSK9 expression, aiming to lower LDL cholesterol and the risk of atherosclerotic cardiovascular disease [29]. The use of LNPs for the delivery of billions of doses of mRNA vaccines during the COVID-19 pandemic established a solid foundation for the safety and commercial viability of LNPs in delivering mRNA genome editors [30]. Still overall, synthetic material-based delivery approaches typically exhibit lower delivery efficiencies when compared to viral-based methods [27]. While additional optimization is feasible, the ultimate efficiency may be constrained by the size and cationic properties of the particles, leading to inadequate dispersion within the interstitial spaces. Target tissues currently are mainly confined to the liver and tumors, where highly permeable vessels allow for the extravasation of delivery carriers into the interstitial space [27]. A relatively new group of biologically inspired nanoparticles, such as virus-like particles (VLPs) and extracellular vesicles (EVs) promise to combine the strengths of both viral-based delivery and synthetic material–based delivery. However, the cost associated with mammalian cell culture and purification may present a limitation to the broad accessibility of these delivery systems.

Due to the limitations of existing delivery methods, new approaches are necessary. Here, we aim to present an initial *ex cellulo* proof of concept for a novel single-layer-synthetic nanoparticle from a non-cationic polymer to package RNP, a particle class that has reached only very limited attention for RNP delivery so far: Nanogels. A hydrogel is a water-expanded polymeric network [31]. A nanoscale hydrogel particle is called a nanogel (NG). They can combine the properties of hydrogels with the capabilities of nanoparticles. Nanogels are highly biocompatible nanoparticles for the delivery of biological loads such as DNA, RNA, or protein [32–36]. Their high water content enables the packaging of hydrophilic biomolecules. Steric inhibition can help protect them from enzymatic degradation or immune recognition. Due to their high surface area for functionalization and tunable physicochemical properties, nanogels can be modified to target specific sites, e.g., by tuning the particle size to exploit the Enhanced Permeation and Retention (EPR) effect when targeting tumor tissue or by adding cell-penetrating peptides to enhance uptake. Nanogels from thiol-functionalized polyglycidol (PG-SH) have been repeatedly shown by our group to be highly biocompatible and capable of active packaging and release of biomolecules over the last decade [34, 36–39]. Here thiol groups form stable disulfide bonds during nanogel synthesis. Recently, they have been used to target tumor tissue in vitro and in vivo after i.v. administration [36, 39], as an antifungal drug delivery system against Aspergillus fumigatus in vitro [40, 41], and to deliver the anti-diabetic peptide RS1-reg to enterocytes in vivo after oral administration [42, 43]. In the latter case, PG-SH nanogels were shown to protect the cargo peptide from degradation in acidic gastric juices. In this study we hypothesized that PG-SH nanogels can entrap Streptococcus pyogenes (S.p.) Cas9-RNP effectively.

Nanogels resulting from PG-SH cross-linking are stable in oxidizing environments but are degraded in reductive environments such as the cytosol. To simulate this stimulus responsive release, we employed a novel cytosol-derived cleavage assay. Most classical RNP activity assays involve incubation of RNP with target DNA followed by agarose gel electrophoresis [3], which is a simple, cheap, and reliable cell-free assay but is also inherently discontinuous, lacks scalability, and has poor sensitivity. Continuous activity assays for different endonucleases exist mainly through (de)quenching of a fluorescence signal following the cleavage of a modified DNA molecule [44]. Efforts to adapt this strategy to Cas9 have struggled because Cas9 remains tightly bound to the DNA after cleavage [45], requiring a protein denaturation step before the final readout [46]. More recently, Marshall et al. have shown that an *E. coli* cell-free transcription-translation (TXTL) system can rapidly and scalable characterize CRISPR nucleases and guide RNA [47]. Here, the readout is decoupled from DNA cleavage via transcription and translation. Since it is derived from bacterial cytosol, we hypothesized that this system could simulate the reductive release of Cas9 while observing nuclease activity in real-time.

## Results and discussion

### Particle characterization

**Fig. 1:**
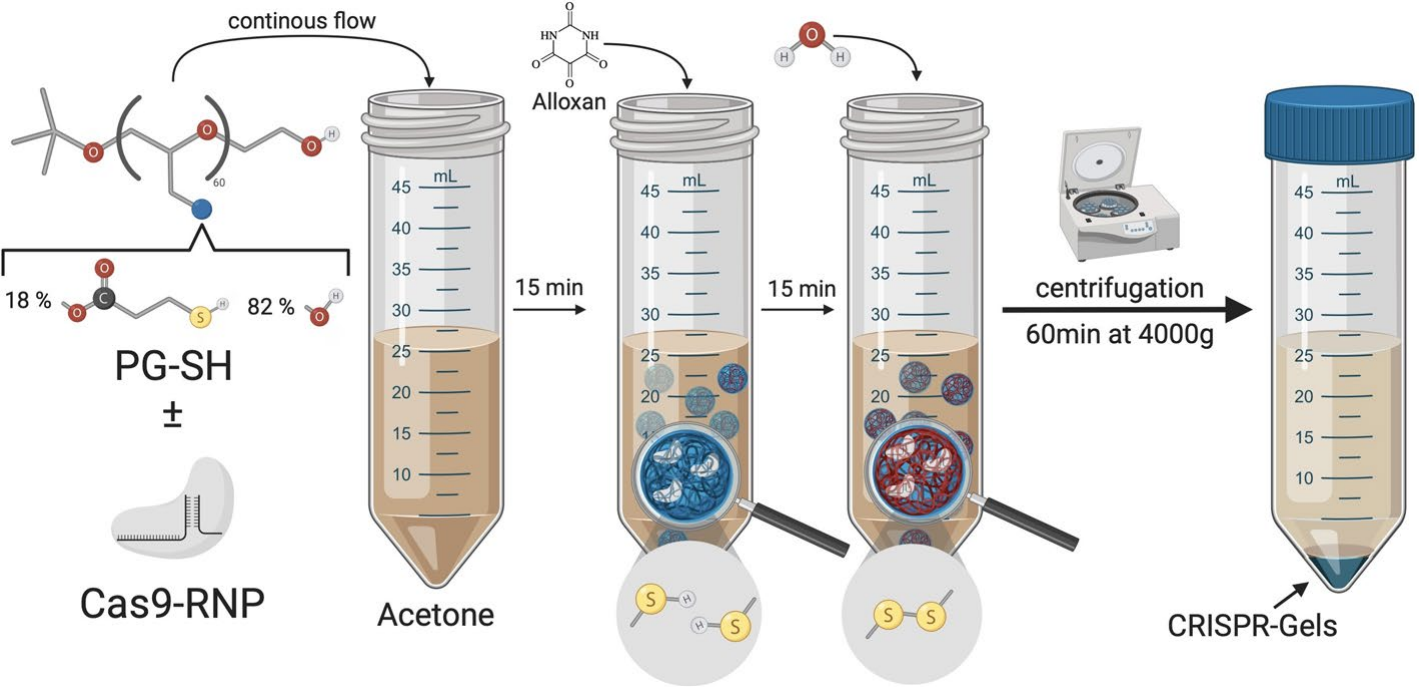
Nanogel synthesis via inverse Nanoprecipitation

CRISPR-Gel synthesis was performed via inverse nanoprecipitation in acetone, as it works without surfactants and high energy input, it is an excellent method for the packaging of enzymes and other labile biomolecules. Figure 1 gives an overview of the synthesis process, for a detailed description see below. Briefly, aqueous PG-SH/RNP solution was mixed with acetone (in excess). Next the pre-polymer was oxidized to form nanogels. Acetone was removed by centrifugation, and CRISPR-Gels were washed and reconstituted in water. Empty and loaded nanogel synthesis was performed in triplicates each sample was analyzed via DLS and NTA as described below.

**Fig. 2:**
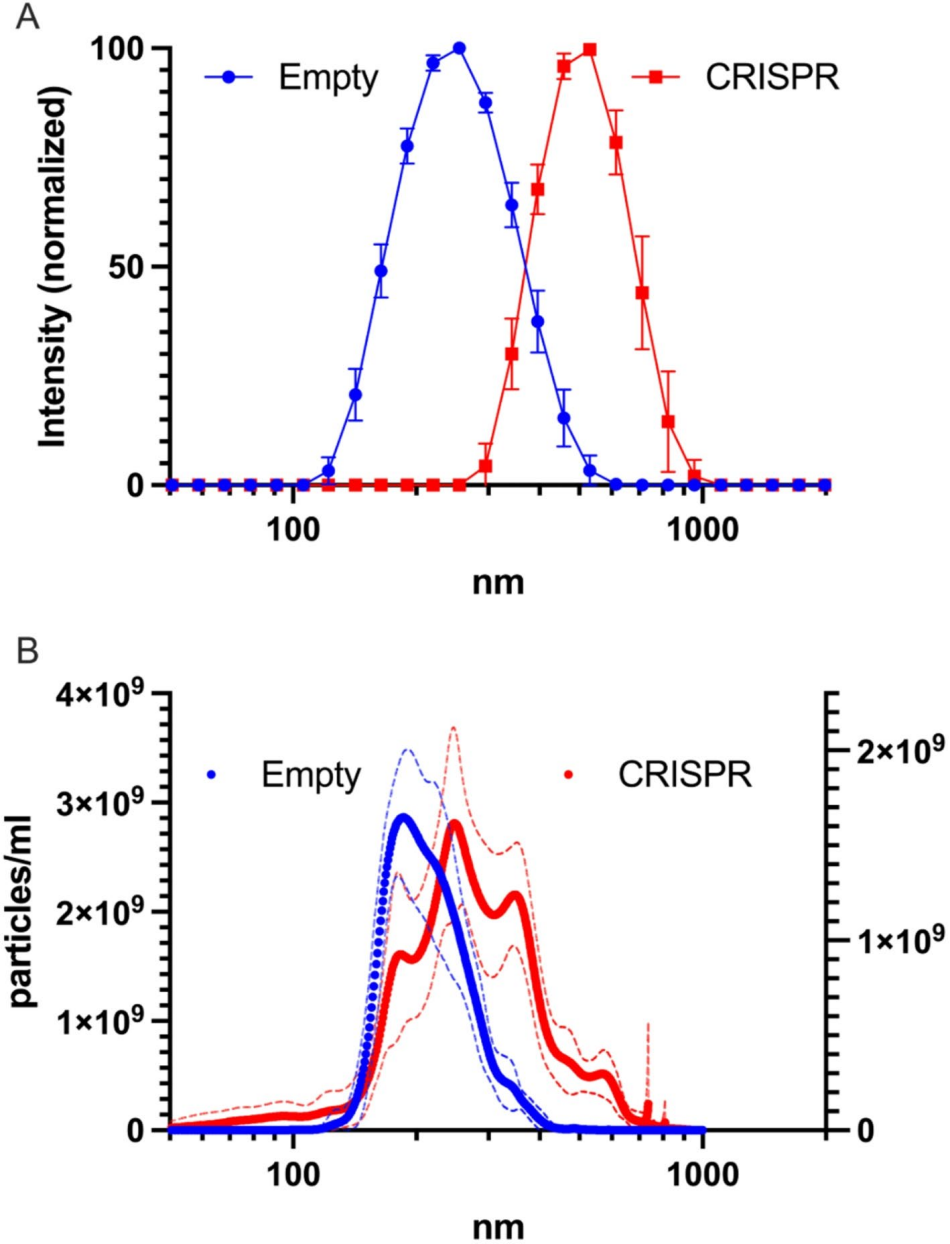
Characterization of empty nanogels (blue) versus nanogels loaded with CRISPR-Cas9 RNP (red).** A** DLS, mean normalized intensity weighted size distribution.** B** NTA, size distribution, dotted lines indicating the standard deviation from the mean

CRISPR-Gels loaded with S.p. Cas9-RNP targeting GFP plasmid (see below) were larger than empty Nanogels. The mean z-average determined by DLS was 239 ± 3 nm (*n* = 3) for empty Nanogels as compared to 497 ± 10 nm (*n* = 3) for loaded CRISPR-Gels (Fig. 2A). Both showed narrow size-distributions with mean pdi values of 0.08 ± 0.02 (*n* = 3) for empty Nanogels and 0.05 ± 0.03 (*n* = 3) for CRISPR-Gels. No difference in Zeta Potential was observed between loaded and empty nanogels. Empty Nanogels showed a mean ZP of − 25 ± 1 mV compared to − 26 ± 1 mV for CRISPR-Gels.

DLS size measurements were complemented by NTA using finite track length adjustment (FTLA). In comparison, with the DLS measurement, both loaded and empty particles showed a smaller size when measured via NTA: 232 ± 4 nm (*n* = 3) for empty nanogels and 333 ± 7 nm (*n* = 3) for CRISPR-Gels (Fig. 2B). In NTA, CRISPR-Gels showed a broader size distribution than empty nanogels. Similarly, high concentrations were achieved for empty nanogels and CRISPR-Gels: 3.4 × 10^11^ ± 4 × 10^10^ and 3.7 × 10^11^ ± 5 × 10^10^ particles/mL, respectively. It should be noted that DLS inherently requires high particle concentrations to generate sufficient scattering intensity for accurate analysis. At such concentrations, particles may aggregate or interact, leading to overestimated hydrodynamic diameters. In contrast, NTA operates at lower, more biologically relevant concentrations, allowing single-particle tracking. NTA analysis of the same samples revealed a smaller mean particle size, which likely better reflects particle behavior under in vitro or in vivo conditions.

The observed increase in particle sizes for RNP-loaded CRISPR-Gels compared to empty nanogels, by both NTA and DLS can be attributed to several factors. Firstly, the addition of RNP in the polymer network before crosslinking could increase mesh size, electrostatic and hydrophobic interactions between the negatively charged RNPs and the nanogel polymers might play a role. Additionally, the hydrophilic nature of RNPs might cause the nanogel matrix to swell during nanogel synthesis. Furthermore, impurities present in the RNP solution, such as ions or glycerin from Cas9/cr-tracrRNA buffers, may interact with the pre-polymer before crosslinking, potentially influencing the size of the nanoparticles. These impurities could alter the polymer’s conformation or crosslinking density, leading to higher particle size. Additionally, differences in the aggregation behavior of nanoparticles with or without RNP loading may contribute to the observed size disparities.

### Reductive degradation

Assessing the degradation of nanogels is important for application in vivo, particularly in the realm of targeted drug delivery. CRISPR-Gels were designed to be stable in the extracellular space and be degraded by the reductive milieu of the cytosol releasing their cargo. Understanding the degradation kinetics of nanogels is crucial for evaluating their stability in the circulation. On the other hand, biodegradability of nanogels is a prerequisite for their safe and sustainable utilization in vivo, ensuring that they can be metabolized and cleared from the body without accumulation thereby causing adverse effects.

**Fig. 3:**
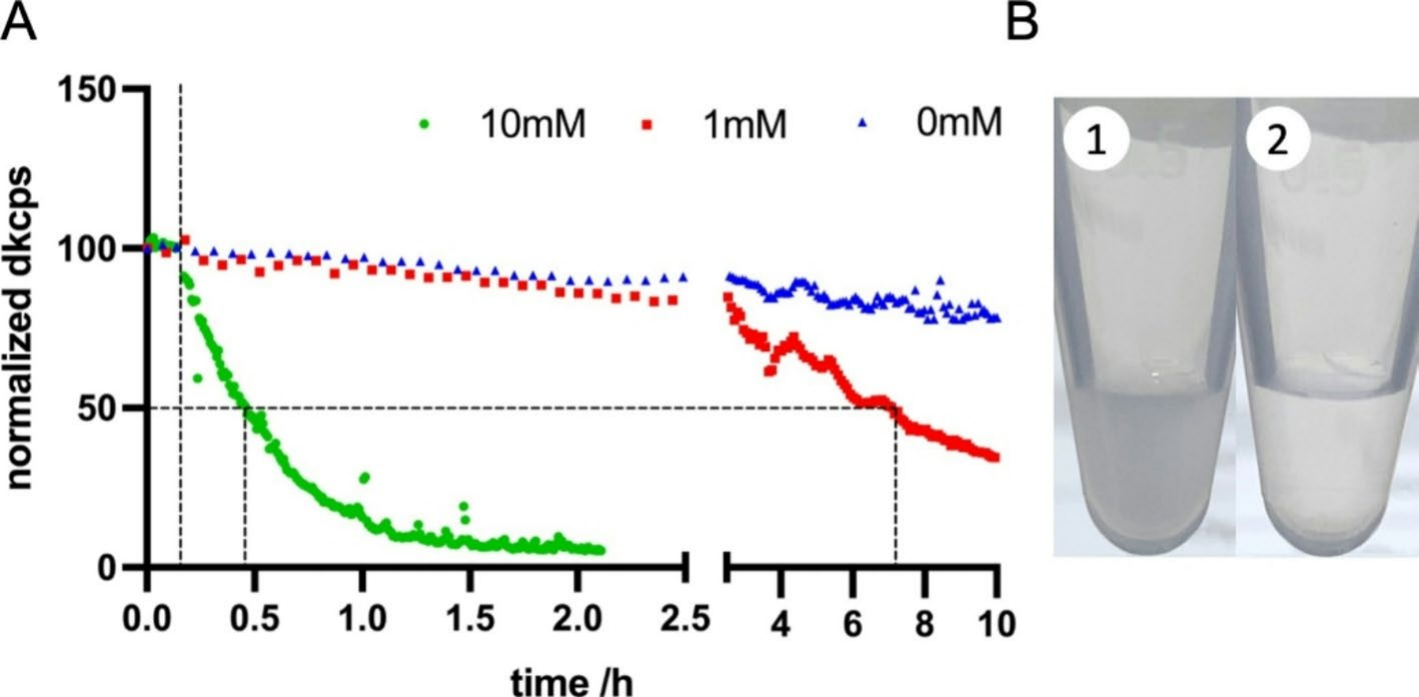
**A**: real time DLS nanogel degradation. To empty nanogels in OPTI-MEM, after 10 min of equilibration (vertical dashed line), Glutathione (GSH) was added to a final concentration of 10mM (green/point) and 1mM (red/square). The blue/triangle dataset was obtained by incubating empty nanogels in DMEM with 10% FCS without GSH. **B**: Macroscopic degradation. (1) concentrated nanogels in water, appearing white due to light scattering (2) clear solution of degraded nanogels 4 min after addition of DTT to 100mM at pH 8.0 in 1XPBS. dkcps: derived kilo counts per second normalized to 100%

A high concentration of nanogels in water appears white due to light scattered (mainly Mie scattering) by the nanoparticles. When the colloid is turned into a solution by reducing disulfide bonds inside the nanogels, it should again be clear, as light scattering decreases dramatically with particle size. Macroscopic degradation was performed in 1.5 mL reaction tubes as described below, 4 min after the addition of DTT at 100 mM and pH 8.0 the turbid colloid (Fig. 3B1) turned into a clear solution (Fig. 3B2), indicating complete degradation.

A different approach is needed to observe degradation in low concentrations of CRISPR-Gels, as light scattering is much less visible macroscopically at lower concentrations. DLS detects the number of scattered photons using Avalanche Photodiodes (APD). The number of photons detected per second is reported as the “count rate.” The minimum count range for viable measurements is in the range of 10^4–5^ counts per second, i.e., 10–100 kilo counts per second (kcps). This rate is adjusted to “derived kilo counts per second” (dkcps) for laser intensity correction. GSH concentration-dependent reductive degradation could be observed in continuous DLS analysis (Fig. 3, A) using the count rate as a surrogate parameter for particle concentration. At 10 mM GSH (37 °C), the count rate halved 17 min after the addition of GSH, and the detection limit of DLS was reached after 126 min. At 1 mM GSH (37 °C), the count rate halved after 414 min, and after 10 h of incubation with 1 mM GSH, ~ 30% of the initial count rate was reached. CRISPR-Gels incubated at 37 °C without GSH showed a count rate reduction to ~ 80% after 10 h. A steady decrease in the count rate might be due to sedimentation or degradation of particles, for the 10 mM GSH we infer to observe mainly particle degradation since a strong concentration dependence could be observed compared to the negative control. For 1 mM / 0 mM sedimentation might contribute increasingly over time. These experiments confirm that degradation occurs at glutathione concentrations within the physiological cytosolic range (1–10 mM). Comparable reductive degradation at physiological pH was also observed for related nanogel formulations synthesized via precipitation as described in our prior work [42]. The time-resolved decrease in scattering intensity correlates with the delayed onset of RNP activity in TXTL reactions described below, supporting the relevance of the degradation kinetics to intracellular conditions.

### RNP loading efficiency

**Fig. 4:**
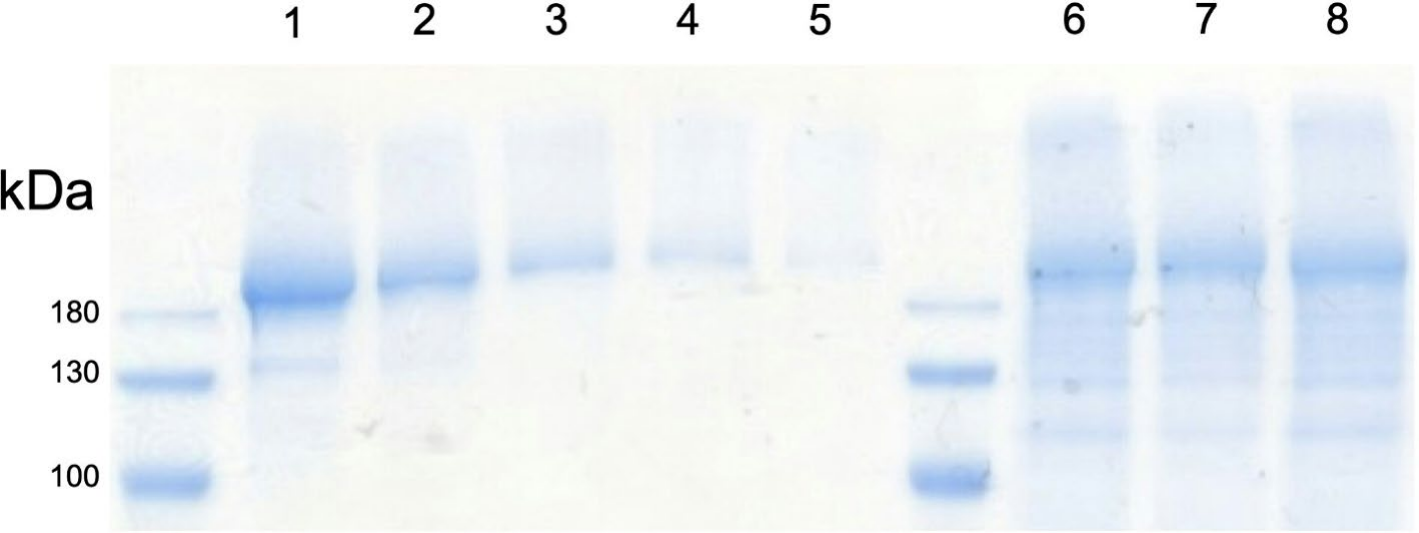
SDS-PAGE with Coomassie brilliant blue staining from a concentration series of RNP starting at 1 µM (lane 1) corresponding to 100% Cas9 retention and subsequent 50% reduction for each lane (lane 2–5). Lanes 6–8 contain CRISPR-Gels, each stemming from individual synthesis

To assess how much RNP was lost during the CRISPR-Gels synthesis process, we performed gel-electrophoresis from CRISPR-Gels that were reductively degraded (s. Fig. 4). Information about the structural integrity could be obtained by comparing the electrophoretic mobility of released RNP to the mobility of unpackaged RNP. Before electrophoresis, all samples were reduced using 100 mM DTT as described below, ensuring full release of all RNP from nanogels. We defined encapsulation efficiency as the relation of total input Cas9 to total Cas9 in the resulting nanogel solution. We achieved encapsulation efficiencies of 60 ± 2% (SD). Importantly, SDS-PAGE analysis of RNP released from fully reduced CRISPR-Gels (Fig. 4, lanes 6–8) revealed a dominant band matching the electrophoretic mobility of pure, unencapsulated Cas9-RNP standards (lanes 1–5), indicating that the protein remains intact and is not truncated. Although lower-molecular-weight fragments are present, likely reflecting partial degradation during synthesis, the preservation of the main protein band suggests successful protection of the majority of the RNP. The presence of a strong, intact Cas9 band in SDS-PAGE after nanogel degradation also suggests that the protein remained soluble and non-aggregated. If Cas9 had undergone significant denaturation during exposure to the organic solvent phase, its hydrophobic domains would likely have become exposed, leading to aggregation and precipitation upon aqueous reconstitution. This would have prevented its detection in the gel. Thus, the observed electrophoretic profile supports not only structural preservation but also the retention of protein solubility.

### Continuous RNP-activity assay via cell-free TXTL

**Fig. 5:**
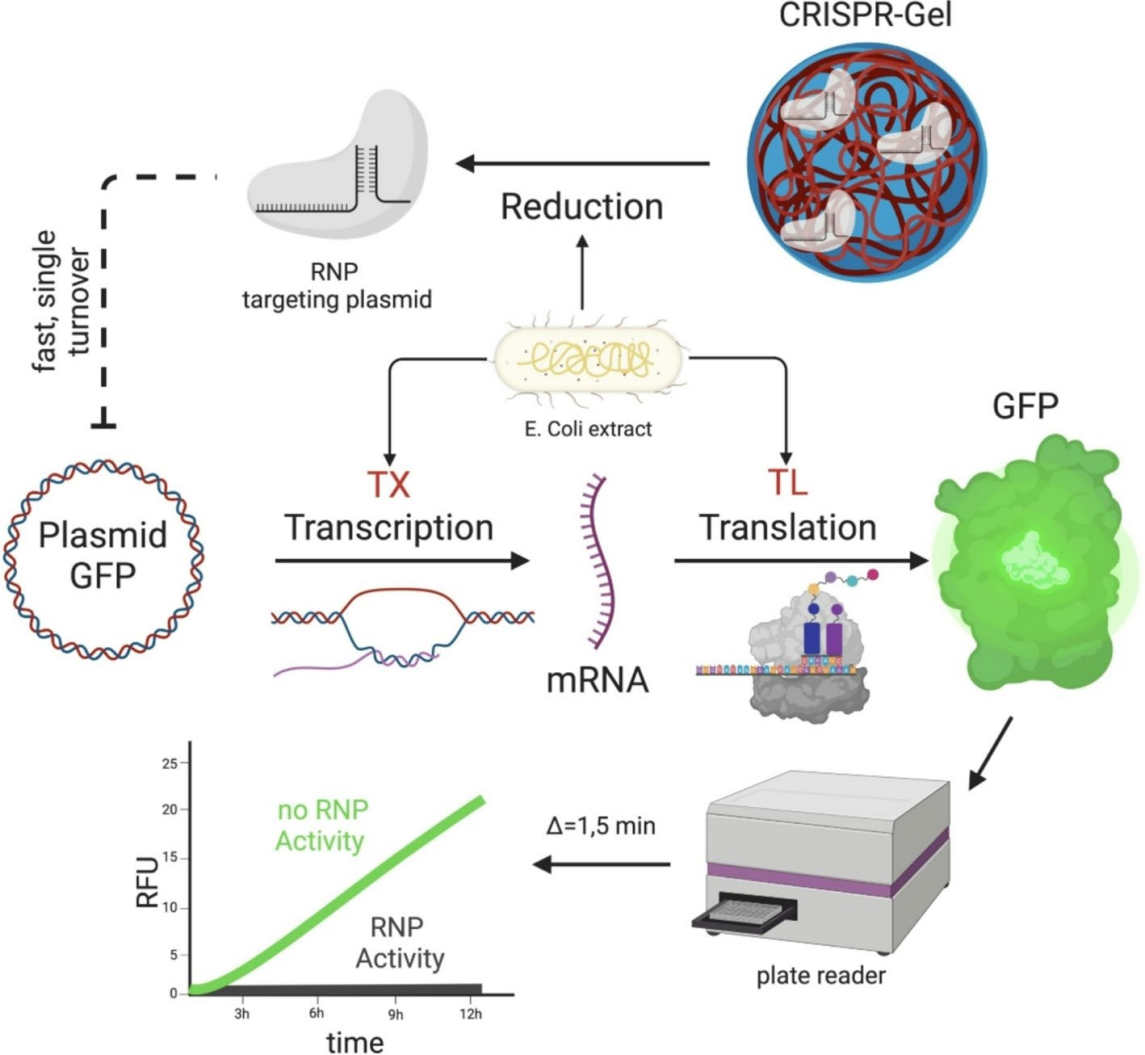
continuous RNP-Activity assay via cell free TXTL. Description: s. Material and methods

TXTL reactions were used to assess the RNP activity from iNPr-CRISPR-Gels. Without RNP-activity, fluorescence increases linearly over several hours, due to stable expression through the TXTL machinery from the plasmid. When RNP cleaves it, expression from the plasmid halts due to direct inhibition of transcription, as the used guide RNA targets the GFP coding region. After cleavage, Exonuclease V, contained in the TXTL mix, will digest the plasmid. Figure 5 gives a schematic overview of the cell-free TXTL assay.

**Fig. 6:**
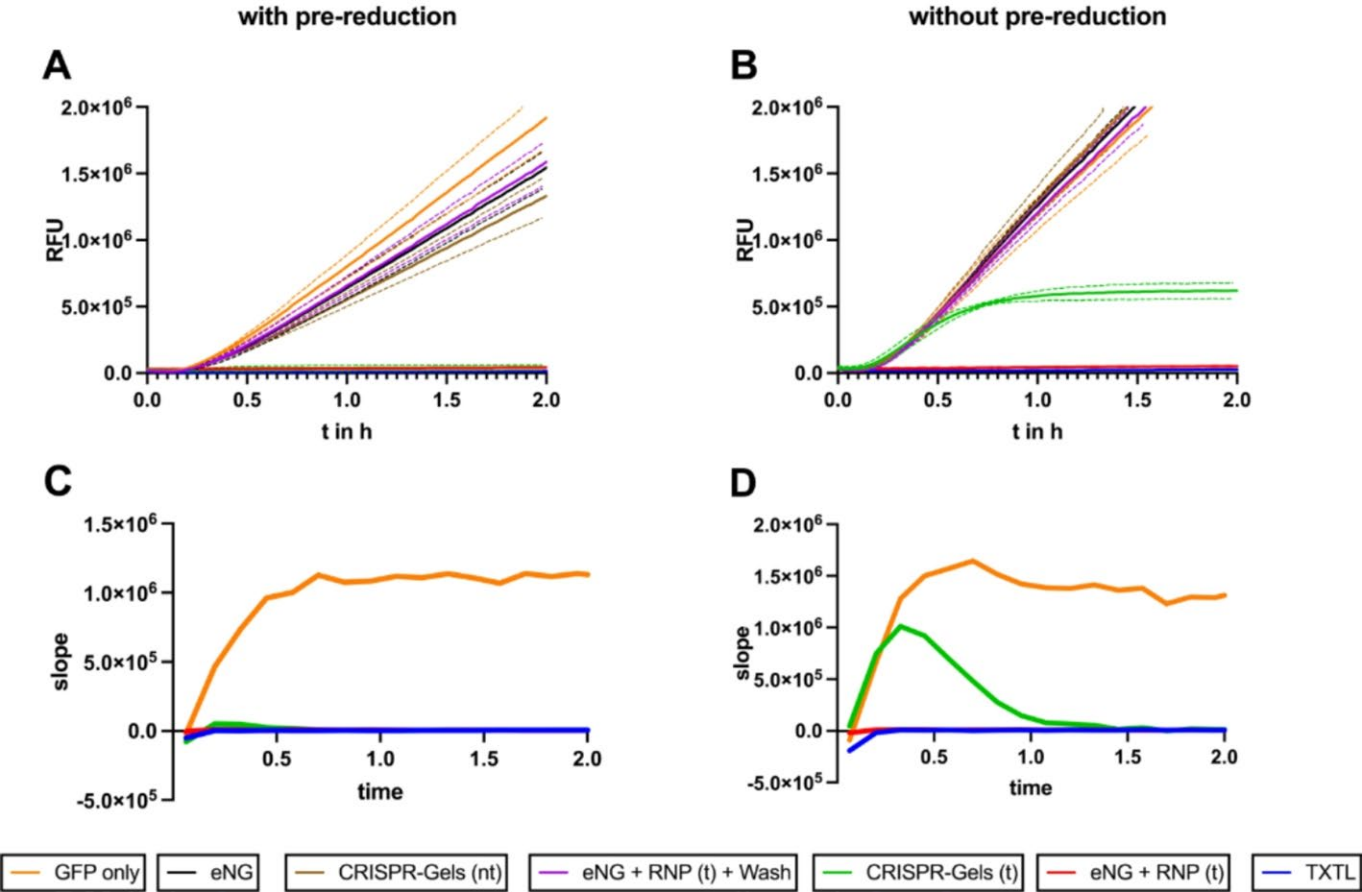
Release of RNP in a cytosol-mimicking environment and real-time cleavage assay.** A**,** B**: Mean fluorescence values (RFU) are plotted against the elapsed time (hours, averaged for one plate reading cycle). The dotted lines represent one standard deviation.** C**,** D**: Slopes of fluorescence derivate of the datasets shown in** A**/**B**. Y-axis: the slope of fluorescence in RFU/hour obtained through simple linear regression over chunks of 7.5 min. X-axis: mean time of the data points used for regression.** A**,** C**: Addition to the TXTL reaction after incubation with DTT (100 mM) at 37 °C for 60 min.** B**,** D**: Addition to the TXTL reaction without prior sample treatment. For a full legend description, see Material and Methods

We compared CRISPR-Gels positive and negative controls. Pure TXTL mix (Fig. 6, blue) showed a baseline fluorescence that increased slowly over time. Reactions containing only GFP plasmid showed high GFP expression, reaching linearity after ~ 15–20 min (negative control, Fig. 6, orange). Fold repression (fr) was calculated from mean RFU values at t = 2 h compared to pure GFP plasmid (s. Table 1, Eq. 1).

1 $$\:\frac{RFU\:at\:t=2\:h\:(GFP\:-only)}{RFU\:at\:t=2\:h\:\left(sample\right)}$$

Empty nanogels showed similar activity (Fig. 6, black), proving that empty nanogels do not inhibit TXTL and RNP activity. To confirm guide specificity, we included CRISPR-Gels loaded with a non-targeting RNP directed against an mCherry plasmid. These reactions showed no significant repression of GFP expression (Fig. 6, brown), confirming that activity in the TXTL system is dependent on the guide RNA sequence. Empty nanogels to which RNP was added, post-synthesis, to a final concentration of 1 µM, corresponding to 100% packaging efficiency, showed repression of GFP expression, ~ 2 fold above TXTL baseline (positive control, Fig. 6, red). The two-fold difference between TXTL baseline and positive control might depend on the time RNP takes to bind its DNA target. Autofluorescence of the nanogels and the GFP labeled Cas9 also contributes to lower fold repression values. Another set of empty nanogels with free RNP (1 µM) was subjected to the same washing procedure as in the last step of iNPr synthesis (16.000 g, 1 × 15 min, and 2 × 10 min). TXTL reactions performed from these gels showed no RNP activity, implying the complete removal of free RNP by the washing procedure (Fig. 6, violet).

To validate successful Cas9-RNP encapsulation in reductively degradable nanogels and assess the release kinetics, TXTL reactions were performed with and without pre reduction of the sample using DTT (100 mM) at 37 °C for 60 min. After pre-reduction RNP from CRISPR-Gels would be immediately available for GFP cleavage, if not reduced before the start of TXTL reactions intact nanogels were hypothesized to prevent immediate interaction of RNP and pGFP, and the TXTL reaction would give real-time fluorescence output of the release kinetics. No difference was observed for the above-described (control) reactions between pre-reduced and native samples. Comparing the result from TXTL reactions with targeting CRISPR-Gels, a difference in fluorescence activity could be observed: CRISPR-Gels that were degraded before addition to the TXTL reaction (Fig. 6A, green) showed immediate repression, leading to stable, low fluorescence, comparable to the activity of free RNP with a fold-repression of 44.7 compared to 46.6 for the positive control (Fig. 6A, red). Reactions containing non-degraded (intact) CRISPR-Gels first showed comparable fluorescence to the negative controls, but after about 30 min, the increase in fluorescence activity slowed down, and fluorescence exhibited a comparable slope to the positive control after 60–90 min, resulting in much lower fold repression (4.2). Figure 6D shows the slopes, calculated via linear regression over 5 data contiguous points (7.5 min), the analyzed time span was determined iterative to minimize noise. This kinetic of fluorescence is compatible with the degradation of CRISPR-Gels in the TXTL reaction and, thereby, cargo release. The observed delay of approximately 30–60 min in RNP-mediated repression corresponds well with the timeframes of nanogel degradation observed in the presence of physiological GSH concentrations as described above, further supporting the idea that release kinetics in TXTL resemble cytosolic behavior.

**Table 1:** Fold repression in relation to TXTL with only GFP at t = 2 h (s. Eq. 1)

	Reduced	Native
TXTL	127.0	97.3
eNG + RNP-t	46.6	50.3
CRISPR-Gels-t	44.7	4.2
eNG + RNP-t + Wash	1.2	1.0
eNG	1.2	0.9
CRISPR-Gels-nt	1.4	0.9

## Conclusion

### iNPr nanogel synthesis is an easy, fast and scalable method for RNP packaging

With iNPr, we identified a fast and easy synthesis method for packaging Cas9-RNP in nanogels. The method described by Horvat et al. (2020) [40, 41] was only slightly modified. By replacing the evaporation of acetone with centrifugation of nanogels in acetone, we could speed up the synthesis process while also reducing possible contamination of the nanogel solution. The whole iNPr synthesis process can be completed in a few hours (excluding particle characterization) by simply adding the aqueous PG-SH solution to acetone; the only device needed is a centrifuge. Furthermore, iNPr can be easily scaled up or down by simply changing the volume of solute and non-solute, upscaling is mainly limited by the maximum size of the vessel for centrifugation. Compared to the commonly employed inverse miniemulsion (iME) method [36–38], which is a lengthy and more complex process for nanogel synthesis from PG-SH, taking 3–4 days (1-day synthesis + 3 days dialysis) more prone to timing and measuring errors, the iNPr method stands out for its efficiency and reduced synthesis time, making it more suitable for high throughput experiments.

### Packaging efficiency and mechanism of immobilization

Since protein synthesis is cumbersome and costly, ideal vehicles for protein delivery should achieve high packaging efficiencies. SDS-PAGE analysis of degraded CRISPR-Gels revealed that iNPr synthesis can achieve Cas9-RNP packaging efficiencies in a magnitude generally suitable for RNP delivery (60 ± 2%) [23]. These results support the theory that RNP can be immobilized by nanogels and protected from degradation. The mechanism of immobilization is unknown. We assume RNP to be located inside of Nanogels; if it only adhered on the nanogel surface, e.g. through electrostatic interactions, the addition of RNP after the nanogel synthesis process would result in similar particles as the addition before crosslinking. The TXTL assay disproved this by showing that the washing procedure can remove RNP activity from a mixture of empty nanogels to which RNP was added post-synthesis. In general, thiol groups of cargo molecules could interact with the (pre) polymer during crosslinking and form covalent bonds. The used protein (6xHis-MBP-4xNLS-Cas9-sfGFP) has 4 Cysteine Residues, 2 in the S. pyogenes Cas9 (C80, C574), and two from sfGFP (C50 and C72), MBP does not contain cysteine residues. Previous studies have shown that the poorly conserved cysteine residues from Cas9 are not essential for enzymatic activity [48, 49] and are unavailable for conjugation reactions [20, 48]. The cysteines in sfGFP are located inside the *β*-barrel structure of correctly folded GFP, rendering them not easily accessible for crosslinking; it has been shown previously that sfGFP (in contrast to GFP) does not form disulfide-linked oligomers in an oxidizing environment (Endoplasmatic reticulum) [50]. Thus, it is not likely that the used RNP forms disulfide linkages to PG-SH during nanogel synthesis. Sterical immobilization by the PG-SH mesh of nanogels is the most plausible explanation of RNP immobilization. In this context, the protein loss during synthesis (40%) might be due to structural damage of RNP during the synthesis process, e.g., by diffusion of proteases into the outer layer of nanogels. Compatible with this “protected core” theory, in Fig. 4, several smaller protein bands are visible compared to the input protein solution from recombinant expression. Additionally, RNP from the outermost layers may diffuse into the solution and consequently be lost during synthesis. This loss would not be detectable in Fig. 4 after washing. Secondly, the loss of RNP in the synthesis process might be explained by a limit to the amount of RNP a single Nanogel can hold. During iNPr synthesis, adding the aqueous solution leads to the formation of nanosized aggregates of PG-SH and RNP. The hydrophilic polymer chains might protect the hydrophilic RNP from direct non-solvent contact. Excess RNP could come into contact with acetone, disrupting its tertiary structure and later being lost after the removal of acetone.

In this study, a larger S.p. Cas9 protein was used (6xHis-MBP-4xNLS-Cas9-sfGFP), with a total molecular weight of 237 kDa compared to 160 kDa for normal S.p. Cas9. Common base editors, such as the BE4-Gam, have a molecular weight of approximately 220 kDa, indicating that the encapsulation capability extends to these larger, multifunctional proteins [51]. 

### Release and real-time activity assay in a cytosol-mimicking environment

In the TXTL assay, iNPr nanogels showed that RNP activity can be maintained throughout the synthesis process. With the TXTL assay, we chose a novel method for assessing CRISPR/Cas9 activity. Negative controls provided the conditions necessary for the validity of the TXTL assay: GFP plasmid was active, empty nanogels did not inhibit the TXTL reaction, activity from released RNP was specific, and the washing process was capable of separating RNP in solution and empty nanogels. The positive controls containing empty nanogels and RNP added post-synthesis showed high repression comparable to pure TXTL, which excludes the possibility of inhibition of RNP activity by the presence of (empty) nanogels. Differences in absolute values and fold repression between baseline and eNG can be explained by minimal initial GFP expression and fluorescence from sfGFP coupled to Cas9.f.

RNP activity from intact CRISPR-Gels was observed ~ 30 min after the start of the TXTL reaction, suggesting the degradation of CRISPR-Gels in the assay. The exact composition of the myTXTL® Sigma 70 Master Mix is not known. The mix stems from the bacterial cytosol of the gram-negative bacterium *Escherichia coli (E. coli)*, where a reductive milieu is present predominantly driven by GSH in the mM range [52], during synthesis of the Master Mix buffer exchanges result in a replacement of GSH by DTT to a final concentration of 0.33–3.33 mM DTT, corresponding to a GSH concentration range of 0.66–6.66 mM as DTT provides twice the reducing equivalents per molecule compared to GSH [53]. Reductive cleavage of the polyglycidol network in the TXTL mix leads to the degradation of CRISPR-Gels and the subsequent release of RNP, bringing the TXTL to a halt. Thus, GFP expression acts as a surrogate parameter for real-time RNP release and activity. Due to the high sensitivity of the TXTL reaction to plasmid concentration, we assume the release of active RNP directly translates into reduced fluorescence slopes. The first slope reduction of reactions with intact CRISPR-Gels, was observed after ~ 30 min, representing the first timepoint RNP activity is measured. Marshall et al. [47] referred to this timepoint as time to repression and observed that it is mainly dependent on the availability of active assembled RNP complex as they saw a shorter time to repression of reactions where Cas9 and guide RNA were pre-expressed compared to reactions without pre-expression. Here, we observed similar results comparing reactions with and without pre-reduction. Nanogel degradation studies with controlled GSH concentrations in the range of cytosolic levels showed kinetics on a similar timescale as time to repression observed without pre-reduction (Fig. 3). These findings support the theory that CRISPR-Gels would be degraded reductively in the cytosol and release active RNP after ~ 30–60 min.

To our knowledge, we describe the first usage of a TXTL mix to mimic the cytosolic reductive release of CRISPR/Cas9 RNP from a nanoparticle. The described assay might also be used to assess the release of RNP from other redox sensible nanoparticles [18, 54]. In contrast to agarose gel electrophoresis-based assays, the native nanoparticles can be directly added to the assay, giving a continuous read-out and allowing for an estimate of the timescale of RNP activity. The TXTL assay was performed in low volume (6 µL) with low variance between replicates, rendering the method suitable for high throughput screening of possible particle candidates. The used myTXTL® master mix is readily available from commercial suppliers but can be synthesized in a cost-effective way from bacterial lysate (~ 0.3$ per reaction) [53]. 

While the TXTL assay provides a powerful tool for assessing Cas9-RNP release and activity under reductive conditions, it remains an artificial system derived from *E. coli* lysate. As such, it does not fully replicate the complexity of the mammalian cytosol, particularly regarding enzymatic degradation, intracellular trafficking, or compartmentalization processes such as endosomal escape. However, the reducing environment of the TXTL mix effectively simulates cytosolic redox conditions. The observed delay in repression kinetics aligns with the degradation timeframes of CRISPR-Gels under physiological reducing agents, supporting the relevance of the model. Despite its limitations, TXTL offers a continuous, high-throughput, and scalable assay format that is particularly well-suited for early-stage screening of redox-responsive nanoparticle systems.

### Prospect: in cellulo validation of CRISPR-Gels

Here, we present the first in vitro, *ex cellulo* proof of concept for PG-SH nanogels as delivery vehicles for Cas9-RNP. Further investigations are necessary to evaluate in vitro and in vivo biological performance of the described system. In general, after systemic administration several obstacles must be overcome: First and foremost, the vehicle needs to be cyto and biocompatible and stable enough to reach its target cell. After entry the vehicle must prevent opsonization and immune recognition, to then extravasate from the blood vessel and pass efficiently through the interstitial space. After endocytosis, the vehicle must effectively release RNP in the cytosol.

Our group has consistently demonstrated the high biocompatibility of PG-SH nanogels in both cell culture and in vivo [36–40, 42]. This high biocompatibility can be partially attributed to their high water content and the hydrophilic polymer composition, which mimics the extracellular matrix. Moreover, reductive degradation into low molecular weight pre-polymers that can be easily excreted via renal clearance prevents accumulation in the organism. While prior studies have established the high biocompatibility of PG-SH nanogels, the current work does not include cellular data. This will be addressed in future investigations.

PG-SH nanogels demonstrated stability in DMEM with 10% FCS over several hours in real-time DLS degradation experiments. These results do not confirm whether the RNP cargo remains encapsulated, as partial reduction of disulfide bonds may suffice to release the cargo before complete particle disintegration. However, the TXTL in vitro release assays indicated that even in a reductive environment, the cargo remains within the nanogels for at least 20 min, a duration potentially sufficient for circulation to the target organ.

A recent case involving AAV gene therapy for Duchenne’s Muscular Dystrophy resulted in death due to severe acute respiratory distress syndrome, caused by an immune response to the AAV capsid. This underscores the need for non-immunogenic delivery systems. The polyglycidol network is biologically inert and will most likely not present an epitope for immune recognition. As pre-formed humoral and cellular immunity against Cas9 protein exists in more than 50% of the population, protecting the cargo from opsonization and immune recognition is crucial as well [55, 56]. According to our protected core theory described above, RNP located in the outer layers of nanogels is lost during the synthesis process; however, its localization deeper inside the nanogel mesh could protect it from antibody diffusion and adherence.

Cellular uptake of nanogels could be enhanced by functionalizing CRISPR-Gels with cell-penetrating moieties such as the TAT (trans-activating) protein derived from the human immunodeficiency virus (HIV). A modified TAT peptide containing a C-terminal cysteine (TAT-SH, CRKKRRQRRR) can be covalently bound to the PG-SH network during CRISPR-Gel synthesis. PG-SH nanogels modified with TAT-SH have been shown to successfully enable miRNA delivery, mediating gene downregulation in multiple myeloma cells and delivering the antidiabetic peptide RS1-reg to enterocytes in vitro and in vivo (mouse model) after oral administration [36, 43]. While uptake is necessary, it is not sufficient for RNP delivery into the nucleus. Our findings indicate that once localized in the cytosol, nanogels can be degraded to release their cargo. The exact mechanism of TAT-mediated uptake of PG-SH nanogels remains unknown. Recent data suggest an endocytic uptake mechanism [42]. Endocytosis typically results in localization inside early endosomes (pH ~ 6.3), which mature into late endosomes (pH ~ 5.5) and eventually fuse with lysosomes (pH 4–5). For effective delivery, nanogels must escape this pathway, a challenge common to non-viral delivery vehicles. Further studies should address endosomal escape, possibly through particle functionalization or tuning particle properties [57].

Additionally, RNP delivery should target specific tissues or cell types relevant to disease pathogenesis. Besides direct injection nanoparticles can target specific organs by taking advantage of “passive” mechanisms such as accumulation in the the liver, limiting venous drainage after i.v. injection or the enhanced permeability and retention (EPR) effect when targeting solid tumors. Ideally, tissue specificity can be “programmed” in nanoparticle attributes to convey “active” tissue specificity. Zilkowski et al. found that subtle changes in the side-chain chemistry of the polyglycidol pre-polymer or fluorophore labeling are sufficient to change the in vivo distribution of nanogels in a murine breast cancer model [39]. This might enable organ targeting inherent in the nanoparticle composition similar to a system recently developed by incorporating different lipids in LNP [58]. Further nanogels provide a high surface area available for functionalization e.g. with antibody (fragments) [59] or receptor ligands [60] offering a separate approach for cell-type specificity that has also shown to be viable for LNP. Additionally, particle size has been shown to play crucial a role in biodistribution of nanoparticles. Clathrin- and caveolae-mediated endocytosis are mostly restrictive to particles < 200 nm. Particle particle uptake in the 200–500 nm range is generally compatible with macropinocytosis, while. Furthermore smaller nanoparticles (< 200 nm) more readily cross biological barriers (e.g. BBB, tumors) and distribute systemically, but are often rapidly cleared by renal filtration. Larger particles (200–500 nm) exhibit longer circulation times, accumulate in organs like the liver and spleen via the mononuclear phagocyte system but show limited tissue penetration [61]. The particles described in this work, fall into the latter category. Importantly this work represents a proof-of-concept, without optimization toward a specific delivery target. Lower particle sizes may be achievable through formulation adjustments, such as polymer concentration, solvent composition, and the use of stabilizers, depending on the desired application and tissue type.

CRISPR-Gels offer several advantages over conventional delivery systems such as LNPs: they are produced via scalable, one-step synthesis, use biocompatible, inert polymers and avoid electrostatic complexation. The system is non-immunogenic, tunable in size and degradation, and easily functionalized for targeting or imaging. These features make CRISPR-Gels a versatile and orthogonal platform for RNP delivery. Importantly this study just provides a first proof-of-concept for using redox-responsive nanogels to encapsulate and release functional Cas9 RNPs. While the physicochemical characterization and in vitro activity demonstrate feasibility, cellular or in vivo data are required to evaluate cellular delivery and gene editing efficiency. These in cellulo data will be essential for benchmarking against established delivery platforms and assessing the translational potential of CRISPR-Gels.

## Materials and methods

### Polymer synthesis

Thiol-functionalized linear polyglycidol (PG-SH) with 60 repeat units and an ester linkage at the side chain were used as pre-polymers for nanogel synthesis. The polymer synthesis was performed as described elsewhere [37]. Briefly, PG-SH was synthesized via carbodiimide-mediated Steglich esterification between the free hydroxyl groups of linear polyglycidol and 3, 3-dithiodipropionic acid. The product of this reaction is a hydrogel, which is subsequently reduced into PG-SH using tris(2-carboxyethyl)phosphine (TCEP). The PG-SH degree of functionalization (DF) was determined using Nuclear Magnetic Resonance (NMR) spectroscopy to be 18%. PhD candidates at FMZ Laboratory, namely Jessica Brand and Sonja Horvat conducted the polymer synthesis at FMZ.

### Recombinant protein expression

An engineered variant of Streptococcus pyogenes Cas9 modified with Simian vacuolating virus 40 nuclear localization sequence (SV40-NLS) (2x on the C Terminus and 4x on the N Terminus) and superfolder (sf)GFP fused on the C terminus was expressed as described elsewhere [3]. Briefly, the protein was expressed from the plasmid 4xNLS-pMJ915v2-sfGFP plasmid (Addgene Plasmid #88921) with an N-terminal hexahistidine tag and maltose binding protein in 8 L Rosetta II cells in 2x YT-Medium (Yeast Tryptone Medium) for 16 h at 18 °C. Expression was induced at an optical density (OD600) of 0.5 using 0.2 mM IPTG (Isopropyl β-D-1-thiogalactopyranoside). The plasmid 4xNLS-pMJ915v2-sfGFP was a gift from Jennifer Doudna (Addgene plasmid # 88921; http://n2t.net/addge ne:88921; RRID: Addgene_88921) [8]. Following expression, the cells were subjected to cell lysis via ultrasound and lyse buffer containing 500 mM NaCl (Sodium Chloride), 20 mM Tris-HCl pH 8.0 (Tris(hydroxymethyl)aminomethane-hydrochloride), 1 mM TCEP (Tris(2-carboxyethyl)phosphine), and protease inhibitors. Purification was performed as described elsewhere [3]. Briefly, the His-tagged protein was purified via IMAC (Immobilized Metal Affinity Chromatography) using HisTrap HP columns. The buffer composition for the IMAC pool included 500 mM NaCl, 250 mM Imidazole pH 8.0, 40 mM Tris-HCl pH 8.0, 10% Glycerin, 1 mM TCEP, and protease inhibitors. The TEV cleavage step was omitted, creating a larger 6xHis-MBP-4xNLS-Cas9-sfGFP Protein of 237 kDa. The mixture was centrifuged at 20,000 ×g for 10 min, yielding a green pellet. Next, we performed Ion Exchange Chromatography (IEX) using a HiTrap SP (sulphopropyl) HP (high performance) column. Elution was performed using a gradient from 150/300 mM KCl to 1 M KCl. The elution buffer composition included 150–1000 mM KCl, 20 mM HEPES-NaOH pH 7.5, 10% Glycerin, and 1 mM TCEP. Gel analysis was conducted, and fractions were pooled. Next, size Exclusion Chromatography (SEC) was performed using a Superdex 200 10/300 GL column. The sample from the IEX pool was concentrated using an Amicon-4 (MWCO = 30 kDa) and loaded onto the SEC column. The fractions were collected with a buffer composition of 150 mM KCl and 20 mM HEPES-NaOH pH 7.5. Gel analysis was conducted, and fractions were combined and further concentrated using a Vivaspin 2 column (MWCO = 30 kDa). The final protein was snap-frozen at − 80 °C for storage. Dr. Lars Schönemann, Recombinant Protein Expression Facility, Rudolf-Virchow-Center for Experimental Biomedicine, University of Wuerzburg, performed Cas9 Expression and purification. Short-term protein storage was at −20 °C, and long-term storage was at − 80 °C.

### RNP formation

crRNA-tracrRNA was annealed by combining crRNA and tracrRNA in equimolar amounts to a final concentration of 10 µM in Annealing Buffer (pH 7.0, 60 mM KCl, 6 mM HEPES). The solution was heated to 78 °C for 10 min and then 37 °C for 30 min, followed by 15 min at room temperature; the annealed dual-guide RNA was immediately used to form RNP complexes or snap froze in liquid nitrogen and stored at − 80 °C for further use. RNP annealing was performed by adding crRNA-tracrRNA and Cas9 together at equimolar amounts and incubating for 5 min at room temperature. Precautions have been taken to prevent RNAse contamination, such as frequent glove changes, RNAase-away surface treatment, and dedicated RNAse-free plasticware. RNA was acquired from Synthego Corporation, Redwood City, CA, USA, as lyophilized powder and reconstituted in Tris-EDTA (TE) Buffer (0.1 mM EDTA, 10 mM Tris, pH 7.5). Reconstituted RNA was stored at − 80 C and used within 6 months. crRNA, targeting the GFP plasmid was designed as described by Marshall et al. [47] (“sgRNA 9”; GUCGCCCUCGAACUU-CACCU + Synthego modified EZ Linker). crRNA targeting the mCherry plasmid (non-targeting) was newly designed (UGAAGGGCGAGAUCAAGCAG + Synthego modified EZ Linker).

### Inverse nanoprecipitation

Inverse nanoprecipitation synthesis of nanogels was modified from Horvat et al. [40, 41]. Briefly, we solubilized PG-SH in water at a concentration of 15 mg/*µ*L; complete dissolution was facilitated by vortexing or, if loaded with cargo, by gently shaking. RNP-loaded nanogels were synthesized by adding RNP (6xHis-MBP-4xNLS-Cas9-sfGFP annealed with crRNA-tracrRNA) to the aqueous PG-SH solution without changing the polymer concentration. 90 µL of the PG-SH solution was added to 45 mL of acetone through continuous pipetting from an electronic pipette (Eppendorf Xplorer® 5–100 µL, Speed: 1). After 15 min, 480 µg of Alloxan was added at a concentration of 32 mg/mL. After 15 min 4 mL of water was added. Next, the acetone was removed from the solution by centrifugation of the acetone/water solution for 60 min at 4000 g. Next, nanogels were washed via centrifugation at 16.000 g, once for 15 min and twice for 10 min. After the nanogels were reconstituted in water, this step was facilitated by three short microtip sonication pulses (amplitude 10%, sonication length/pulse 0.4 s, and pause 0.6 s). Sonification was performed cooled inside an aluminum microtube rack on ice.

### Dynamic light scattering (DLS)

This study used the Zetasizer® Nano ZSP by Malvern Analytical to perform DLS. Samples for size measurement were prepared for DLS by diluting concentrated particle solution 1:100 in water directly after spin purification. The sample was loaded in disposable plastic micro cuvettes (ZEN0040, Malvern Panalytical Ltd). Dispersant viscosity (Water at 25 °C) was assumed to be 8872 cP, and Refractive Index (RI) was assumed to be 1.330. Particle material was set to “Protein” with a RI of 1.450 and Absorption of 0.001. The narrow band filter was not activated. Measuring duration, position, and attenuation was set automatically by the software for each measurement. Each sample was analyzed four times in a row; each measurement consisted of at least 10 runs, collecting scattered light over 10 s each run. Measurements were performed at 25 °C with 120 s equilibration time. For Zeta Potential analysis, samples were transferred from the cuvette used for size analysis to a DTS1070 cell (Malvern Panalytical Ltd) disposable folded capillary made of polycarbonate with built-in gold-plated copper. Dispersant constants (RI, Temperature, Viscosity) were assumed to be the same as for size measurement, the dielectric constant was set to 78.5. Particle viscosity was assumed to be sample viscosity, and particle material was set to protein. The Smoluchowski model was used with an F(Ka) value of 1.50. The Zetasizer software automatically determined measurement duration, Voltage, and attenuation. Each sample was measured at least 10 times, consisting of 10–100 runs. The narrow band filter was not activated. The software used was Zetasizer 7.11 (Malvern Panalytical Ltd).

### Nano tracking analysis (NTA)

In this study, NTA was performed using a Nanosight NS300 by Malvern Panalytical; it operates within the ISO standard. Samples were prepared by dilution with water. The optimal dilution rate was determined by dilution series and choosing a dilution for which each frame contains 20–100 particles; in general, particles after the last purification step were diluted 1:10^3^. Samples were loaded in 1mL sterile, single-use, low dead space, polypropylene syringes with Luer slip connector (Injekt®-F 1 mL, B. Braun, Germany). We used the low-flow cell chamber (LVFC) supplied with the NS300. The chamber temperature was set to 25 °C (target tolerance 0.1 °C for ≥ 5s), the camera level was set to 10, the focus was set manually, and the detection threshold was set to 5. The sample was infused at a continuous flow rate of 40 (AU) using the NanoSight syringe pump (Malvern Panalytical Ltd.) for 30 s before the first measurement. Six individual videos over 60 s were obtained, and continuous flow was provided to increase the number of analyzed particles and improve statistic strength; the resulting mean particle drift (~ 2.5 pixels/frame) was calculated by the software in real-time and deducted during analysis. Before the next sample the system was flushed using at least 2 mL water at a pump speed of 750. The device’s software calculated the diffusion coefficient and size distribution.

### Nanogel reduction

To assess macroscopic degradation by reduction light scattering from a nanogel-colloid, to 35 µL of concentrated nanogels in water (2.355 × 10^11^ particles per mL), 10 µL Dithiothreitol (DTT) was added to a final concentration of 100 mM, pH was set to 8.0 using 5 µL of 10X PBS. The experiment was done at room temperature, ~ 25 °C. Video footage was obtained using the front camera of an iPhone 13 Pro. In the background, a white piece of paper with black text out of focus is visible. Pictures in Fig. 3, b were cropped and equally adjusted for contrast and white balance.

For real-time DLS degradation by count rate the initial concentration at t = 0 min was 4.45 × 10^9^/ mL. All datasets were normalized, and the first value was defined as 100%. The dispersants were HEPES buffered at pH 7.5. All measurements were performed at 37 °C using the heat conducting cap on top of the cuvette to facilitate uniform heat distribution. The count rate is measured in real-time on the scale of nanoseconds. In the Zetasizer Software, the real-time count rate data is not accessible. The Zetasizer offers a “count rate meter,” designed to observe sample quality before the experiment; it displays the count rate but offers no record function. Alternatively, a SOP was created, performing many repetitive short size-measurements. A 5s minimum pause was required between measurements, defined by the minimum time needed to exchange cuvettes for adding the reductive agent, as without a cuvette, the measurement would abort. Time was extracted using a Python script as reported by Zetasizer Software (rounded to full seconds). The measurement was continued for 10 h or until the count rate reached low values in the range of 10^5^. Three data points in Fig. 3 were censored from the 10mM dataset, as the sample was not inside the machine during the addition of GSH. The Blue and red datasets were acquired with a 5-minute delay between measurements. No censoring was necessary here.

### SDS-PAGE

SDS polyacrylamide gel electrophoresis (5% stacking gel and 10% separating gel) was performed as described by Laemmli et al. [62] in a Mini-PROTEAN® II slab gel apparatus (Bio-Rad, Hercules, California, USA) for 60 min at 200 volts. For standard sample preparation, the samples were pretreated for 30 min at 37 °C in 60 mM Tris-HCl, pH6.8, 100 mM dithiothreitol, 2% (w/v) SDS, and 7% (v/v) glycerol and then separated by SDS-PAGE. The gels were stained with a colloidal coomassie brilliant blue staining solution, according to Candiano et al. [63]. After inverse Nanoprecipitation synthesis, CRISPR-Gels loaded with 350 pmol RNP (6xHis-MBP-4xNLS-Cas9-sfGFP, 236,8 kDa annealed with crRNA-tracrRNA) per synthesis were reconstituted in 350 µL water. One CRISPR-Gel synthesis was loaded per lane (lanes 6–8, Fig. 4). A dilution series, starting at the concentration of RNP corresponding to a 100% retention rate after iNPr synthesis (1 µM) with 50% concentration reduction for each following lane (lanes 1–5, Fig. 4), was created to obtain a standard curve. Per lane, a 20 µL sample was applied. Densitometric analysis was performed using the ImageJ software. Simple linear regression analysis was employed to establish the relationship between protein concentration and band intensity. Thorsten Keller performed SDS-PAGE.

### Continuous RNP-activity assay via cell free TXTL

The commercially available myTXTL® cell-free transcription and translation master mix was provided by Arbor, Michigan, USA (from here on referred to as “TXTL mix”). It is obtained from *E. coli* lysates and contains all the necessary biomolecules to perform transcription and translation and maintain an energy metabolism fueling these reactions. The detailed composition and characteristics of the mix is described elsewhere [64]. Fig. 5 gives a schematic overview of the TXTL assay. TXTL reactions were assembled, modified from Marshall et al. [47]. Each reaction contained the myTXTL® Sigma 70 Master Mix, deGFP expressing plasmid (pTXTL-P70a-deGFP HP, final concentration: 0.6 nM), and a sample. deGFP fluorescence was used as a surrogate for deGFP expression by the TXTL mix. The reaction was performed in black polypropylene 96 Well Plates with V-shaped wells. The reaction volume was 6 µL, containing 5 µL of TXTL mix, including a GFP plasmid and 1 µL of the sample. Plates were closed using an adhesive plate seal developed for qPCR applications with high optical clarity and minimal autofluorescence. Sample preparation took place on ice only to prevent the premature start of the TXTL reaction. Some samples were incubated in Cas9 Working Buffer (WB) containing 100 mM of DTT at pH 8.0 and 37 °C for 60 min before addition to the TXTL-mix.

Here, eGFP plasmid was added together with the TXTL-mix only after incubation. The fluorescence reading was performed using a TECAN Spark 20 M (Tecan Group Ltd., Männedorf, ZH, Switzerland). The read interval was set to 90 s, and the temperature control was set to 29 °C. GFP Fluorescence was read from the top using a Monochromator: Excitation wavelength: 485 nm, bandwidth 20 nm; Emission wavelength: 535 nm, bandwidth 20 nm. The gain was set automatically by the SparkControl software (V1.2.25). The following settings were set: number of flashes: 30, integration time: 40, lag time: 0, Settle time: 0, Z-Position: manual, 16,000.

Sample description for Fig. 6A–C: Each reaction contained the myTXTL® Sigma 70 Master Mix, deGFP expressing plasmid (final concentration: 0.6 nM), and a sample as follows (n = number of individual TXTL reactions and CRISPR-Gel synthesis). Orange: water. (*n* = 2) Black: empty Nanogels. (*n* = 2) Brown: CRISPR-Gels loaded with active RNP targeting the mCherry plasmid (nt). (*n* = 3) Violet: Empty nanogels to which active RNP, to a final concentration equivalent to 100% retention rate (1 µM), have been added post-synthesis and were rewashed via centrifugation as described in the final step of iNPr synthesis after the addition of free RNP. (*n* = 2) Green: CRISPR-Gels targeting the deGFP plasmid. Red: empty Nanogels to which active RNP has been added at a concentration equivalent to 100% retention rate (1 µM). (*n* = 2) Blue: pure TXTL-mix with water instead of sample and GFP plasmid. (*n* = 1).

## Electronic supplementary material

Below is the link to the electronic supplementary material.

Supplementary Material 1

## Data Availability

The data that support the findings of this study are available from the corresponding author upon reasonable request.
