# Supplementary figures and images for: Efficient encapsulation of CRISPR-Cas9 RNP in bioreducible nanogels and release in a cytosol-mimicking environment
Source: Discov Nano. 2025 Jul 26;20(1):119. doi: 10.1186/s11671-025-04316-5 (PMC12297123; doi:10.1186/s11671-025-04316-5)

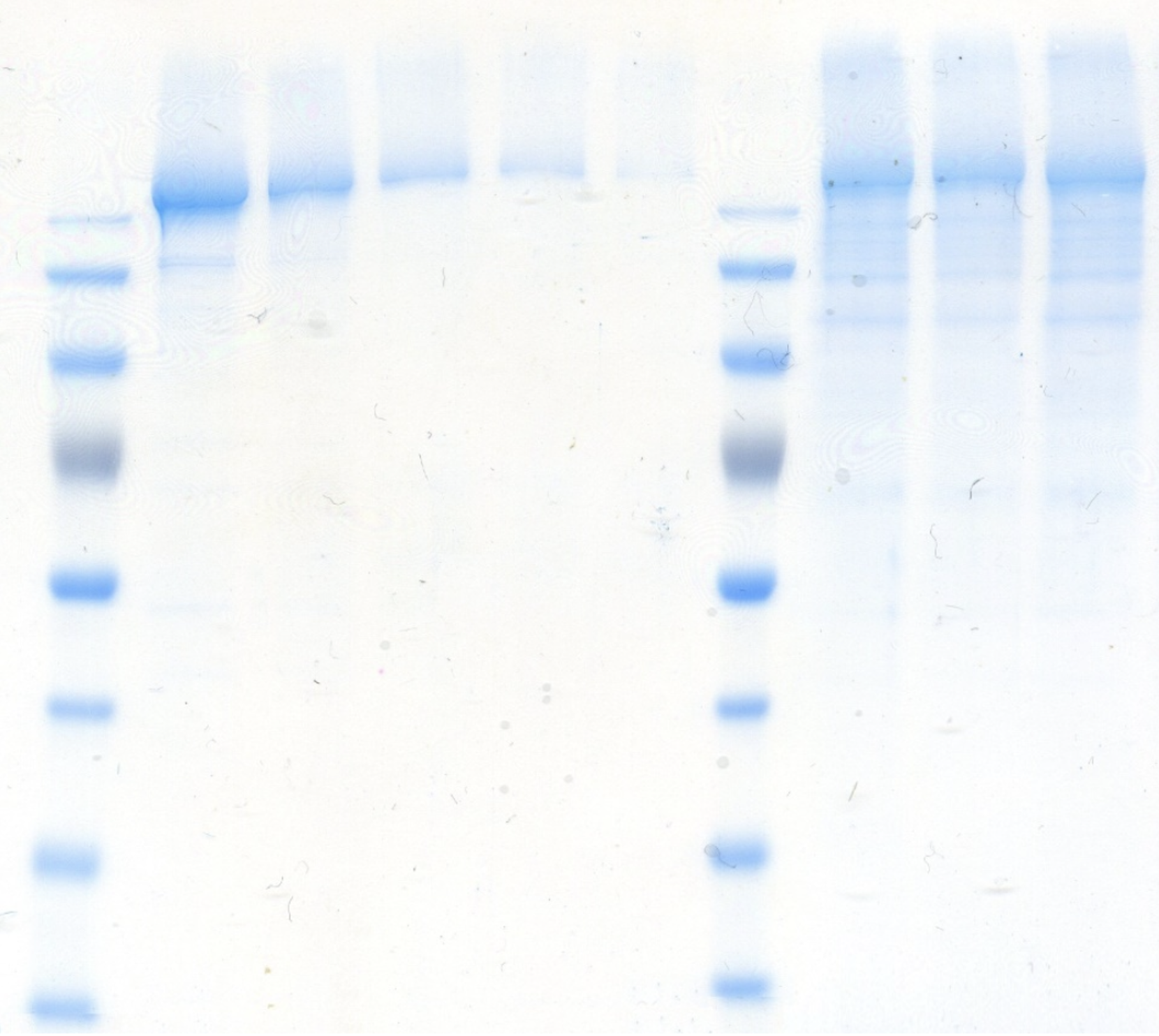

Supplement: Supplementary file 1 — Supplementary Material 1 [file 11671_2025_4316_MOESM1_ESM.png]
